# Comparisons of *Schansitherium tafeli* with *Samotherium boissieri* (Giraffidae, Mammalia) from the Late Miocene of Gansu Province, China

**DOI:** 10.1371/journal.pone.0211797

**Published:** 2019-02-12

**Authors:** Sukuan Hou, Michael Cydylo, Melinda Danowitz, Nikos Solounias

**Affiliations:** 1 Key Laboratory of Vertebrate Evolution and Human Origins of Chinese Academy of Sciences, Institute of Vertebrate Paleontology and Paleoanthropology, Chinese Academy of Sciences, Beijing, China; 2 CAS Center for Excellence in Life and Paleoenvironment, Beijing, China; 3 College of Earth and Planetary Sciences, University of Chinese Academy of Sciences, Beijing, China; 4 Department of Anatomy, New York Institute of Technology College of Osteopathic Medicine, Old Westbury, NY, United States of America; 5 Department of Pediatrics, Alfred I. duPont Hospital for Children, Wilmington, DE, United States of America; 6 Department of Paleontology, American Museum of Natural History, New York, NY, United States of America; Fred Hutchinson Cancer Research Center, UNITED STATES

## Abstract

We are describing and figuring for the first time skulls of *Schansitherium tafeli*, which are abundant in the Gansu area of China from the Late Miocene. They were animals about the size of *Samotherium* with shorter necks that had two pairs of ossicones that merge at the base, which is unlike *Samotherium*. The anterior ossicones consist of anterior lineations, which may represent growth lines. They were likely mixed feeders similar to *Samotherium*. *Schansitherium* is tentatively placed in a very close position to *Samotherium*. *Samotherium* and *Schansitherium* represent a pair of morphologically very similar species that likely coexisted similarly to pairs of modern species, where the main difference is in the ossicones. Pairs of ruminants in Africa, for example, exist today that differ mostly in their horn shape but otherwise are similar in size, shape, and diet. The absence of *Schansitherium* from Europe is interesting, however, as *Samotherium* is found in both locations. While is it challenging to interpret neck length and ossicone shape in terms of function in combat, we offer our hypothesis as to how the two species differed in their fighting techniques.

## Introduction

Giraffidae are Pecora ruminants [1]. There are approximately twenty-five species of Giraffidae, two extant and the rest are extinct [2–3]. The modern giraffe possesses exceptionally elongated cervical vertebrae and metapodials; the extinct taxa exhibit varying degrees of neck and limb elongation [4–6]. Giraffidae possess specialized cranial appendages termed ossicones, which start as separate ossifications that subsequently fuse to the skull [2, 7–9]. Among the Giraffidae, several taxa exhibit atypical ossicone structure, including the sivatheres *Sivatherium* and *Bramatherium* and the samothere *Schansitherium*. The first two have been reported and figured [10–12]. Unlike these, *Schansitherium* is poorly described and studied.

*Schansitherium tafeli* is a giraffid from the late Miocene (Beodian age) of North China [2, 13–14]. Although it is a fairly abundant species in North China and is represented by complete cranial and postcranial material, it has not yet been figured nor studied in detail. It is a species similar to *Samotherium*, which is also found at the same deposits [14]. For example, some of the key similarities are: absence of sinuses in the calvaria, the position of the main ossicone (posterior ossicones of *Schansitherium* and the only pair of ossicones of *Samotherium*), the masseteric fossa size, the basicranium and area surrounding the foramen magnum, the occipital shape, the dentition. *Schansitherium tafeli* was named on an isolated skull by Kilgus in 1922 from a collection of fossils from Shanxi, which is housed in the University Museum of Tübingen in Germany. *Samotherium boissieri* was named on a complete, crushed skull from Samos by Major in 1888 [3, 15–16]. The cranial and postcranial material of *Samotherium boissieri* is housed at the Natural History Museum NHMUK in London. Since 1922, several skulls and numerous postcranial specimens have been attributed to *Schansitherium tafeli* and *Samotherium boissieri* that have been found in North China.

The most recent and statistically supported cladogram of the Giraffidae places *Schansitherium* adjacent and primitive to *Samotherium* [17]. Of the various *Samotherium* species, we chose *Samotherium boissieri* to compare with *Schansitherium tafeli*, based on similarities between the skulls, dentitions and postcranial elements of the two taxa [18–19]. In addition, *Samotherium boissieri* is the most abundant *Samotherium* species in Gansu, where it coexisted with *Schansitherium tafeli* in the Miocene. We provide the first detailed description of *Schansitherium tafeli*, and we compare this taxon to *Samotherium boissieri*.

The studied *Schansitherium tafeli* material was found in the Linxia Basin. The Linxia Basin is located in the transitional zone between the Tibetan and Loess plateaus, which is filled with 700–2000 m of Cenozoic deposits [20–22]. The *Schansitherium tafeli* specimens were excavated from the Upper Miocene Liushu Formation, which consists of mudstones and marls. Four representative Late Miocene faunas are recognized, from older to younger: the Guonigou, Dashengou, Yangjiashan, and the Qingbushan faunas. *Schansitherium tafeli* are abundant in the Dashengou Fauna and Yangjiashan Fauna, dated at about 8–10 Ma.

## Material and methods

We studied *Schansitherium* and *Samotherium* material in Beijing IVPP and Hezheng HPM. We also studied in person the *Samotherium* material in AMNH, NHMUK, and other museums. We describe the skull, third cervical vertebra, metacarpal and metatarsal of *Schansitherium tafeli* from Gansu and of *Samotherium boissieri* from AMNH, NHMUK, and Gansu. The material from AMNH and NHMUK were previously studied by Kostopoulos [23], Hamilton [2], and Bohlin [14]. The material from Gansu are described for the first time in this paper. The *Samotherium boissieri* skull used for the comparisons is of great value as it is complete and not crushed. This skull enables us to study the details of the ossicone and its apex, the occipital and the basicranium. We compare the cranial and post-cranial material of these two taxa. We use cervical vertebral terminology and characteristics established by Danowitz and Solounias [24] and Danowitz et al. [25]. We use metapodial terminology established by Rios et al. [6].

***Schansitherium tafeli***:

Skulls: AMNH 30502 (plaster cast holotype skull from Tübingen); HMV 1740, 0945, 1943, 1931, 1416, 1321, 1934, 1932, 1572

Vertebra: HMV 1988

Metacarpal: HMV 1951

Metatarsal: HMV 1986

***Samotherium boissieri***:

Skulls: IVPP V20167, V20271

Vertebra: C3 –NHMUK 4250

Metacarpal and metatarsal: AMNH 15876

**Institutional abbreviations**

AMNH, American Museum of Natural History, New York, USA. NHMUK, Natural History Museum United Kingdom, London, UK. HPM Hezheng Paleozoological Museum, Gansu, China. IVPP, Institute of Vertebrate Paleotology and Palaeoanthropology, Chinese Academy of Sciences, Beijing, China.

## Description of *Schansitherium tafeli* (Tables 1 and 2 summarize the morphology and size of the specimens)

**Table 1:** Comparisons of *Schansitherium tafeli* with *Samotherium boissieri*.

Character	*Schansitherium* (HMV 1740, 0945)	*Samotherium* (IVPP V20271)
anterior border of orbit	behind M3	middle of M3
post-orbital bar	rotated more caudally	rotated more laterally
masseteric fossa	deeper	shallow
anterior basioocciptal tuberosities	same	same
bulla	round but flat	round, bulbous
post glenoid process	round tongue with long extension laterally	rounded, tongue
posterior border of the palatine bone	V shape and behind M3 (U and behind M3), narrow	wide U shaped, behind M3
ossicone	heavy No.1, four ossicones, compressed (has keel behind the ossicone in HMV 1932)	small base, long ossicone, long distance, direct more posteriorly, end sharp, little No.1, smooth surface with vertical lines
ossicone	ossicone is longer, thinner more abruptly	ossicone is shorter, doesn't thin out
secondary bone growth	descends down the ossicone forming ridges, streaks	smaller, near the apex
ossicone apex	more pointed	rounded knob, small, bulbous
ossicone bumps, outgrowths	cranial and caudally located bony outgrowths on ossicone sometimes	
ossicone	ossicone starts at the superior margin of the orbit	ossicone starts at anterior margin of the orbit
anterior ossicone	short, apex looks distorted with bony growths, bumps	doesn't exist
anterior ossicone	multiple layers (horizontal)	N/A
surpa orbital impressions	spread from the supraorbital foramen	confined
ventral surface of the zygomatic zrch	bumps (HMV 1932 smooth)	smooth
occipital crest	with median depression	no median depression
occipital condyles	more dorsal, at level of suture	extend lower
dorsal foramen magnum growths	not present	present
P2	more *Samotherium* like (HMV 1932 more *Honanotherium* like)	styles thinner,metastyle smaller
cingulum of the premolars	*Samotherium*like (HMV 1932 more *Honanotherium* like)	lingual weak, labial abscent
lingual side of the premolars	round	flat
bone behind M3	medium	like in Hipparion
cingulum of the molars	weak (HMV 1932 absent)	lingual weak, labial abscent or weak
p3	island of enamel	no island
p2	five lingual cuspids	one lingual cuspid

**Table 2:** Measurements of *Schansitherium* and *Samotherium*.

	*Schansitherium tafeli*						*Samotherium boissieri*
Specimen No	HMV 1932	HMV 1740	HMV 1931	HMV 1943	HMV 1934	HMV 0945	IVPP V20271
modified skull length	429	417	410	430	435	463	450
length from behind the ossicone to the occipital crest		173	158				
length from postorbit process to the occipital crest	205	221	ca 215				
length from anterior orbit rim to the tip of the nasal bone	315	-	-				
narrowest part of the parietal crest	80	70.6	73.7				
ossicone width at base	91.5	95	94.5	120	101	119.5	84
ossicone base length	122	120	142				
ossicone length	179	243	195	220	203	238	225
anterior length of the small ossicone		66	99				
posterior length of the small ossicone		40	40				
anterior-posterior diameter of the small ossicone		30	32				
lateral-medial of the small ossicone		19.3	30				
height of the ossicone base	44	49.5	62.5				
width of the orbit	63.4	60	68				
height of the orbit	74	61.5	53				
minimal width of the postorbit process	26	25.8	24.5				
minimal height of the inferior orbit rim	-	6	6				
distance between the ethmoid fissure and the orbit	66	44	ca 50				
width of the ethmoid fissure	49	-	-				
height of the ethmoid fissure	35	-	-				
width of the nasal bone	49	>37	-				
height of the maxilla above anterior of P2	88	86.4	-				
height between the post M3 and the orbit	80	93	-				
distance between preorbit foramen and P2(from the boundary of the crown and the root)	26	34	-				
narrowest point of the sagittal groove on the ventral surface of the maxilla	8.3	2	-				
palatine width in front of P2	67	45	-				
palate width at M1	82	68	95.5				
palate width at M3	83	67	102				
length of palatine bone	60	67	66.5			63	77
length of maxillary bone(from P2 to maxillary-palatine suture)	98	94	101			88	98
width at the widest part of zygomatic	210	185	ca 234				
skull width at M2	146	ca 149	156	158	162	130	152
occipital condyles width	97	100	ca 122	95	130	100	102
ovale length	14	16	15	16		16.5	9
ovale width	9	8.5	10	12.5		11	9
preorbital foramen length	19	21	-	16	22.5	24	27
preorbital foramen width	9	8.5	-	8	9	10.5	7.5
posterior b-tuberosities width	58	62		65	60	51.5	66
anterior b-tuberosities width	35	27			28.5	27	27
snout width at preorbital foramen	106	76.5	96		116	104	95
snout width 4cm before preorbital foramen	92	72.5	-		96		
occipital crest width	143.5	127	157		156	148	121
narrowest part of the occipital region	92.8	111.7	ca 114				
occipital width at the paroccipital base	171.8	167	ca 185				
zen	4	4.5	-				3
P2-P4 length	77	79			82.5	83	86
M1-M3 length	106.3	106.5	105		116.5	111.5	109
P2-M3 length	176.1	192	-				

### Cranial

**Fig 1:**
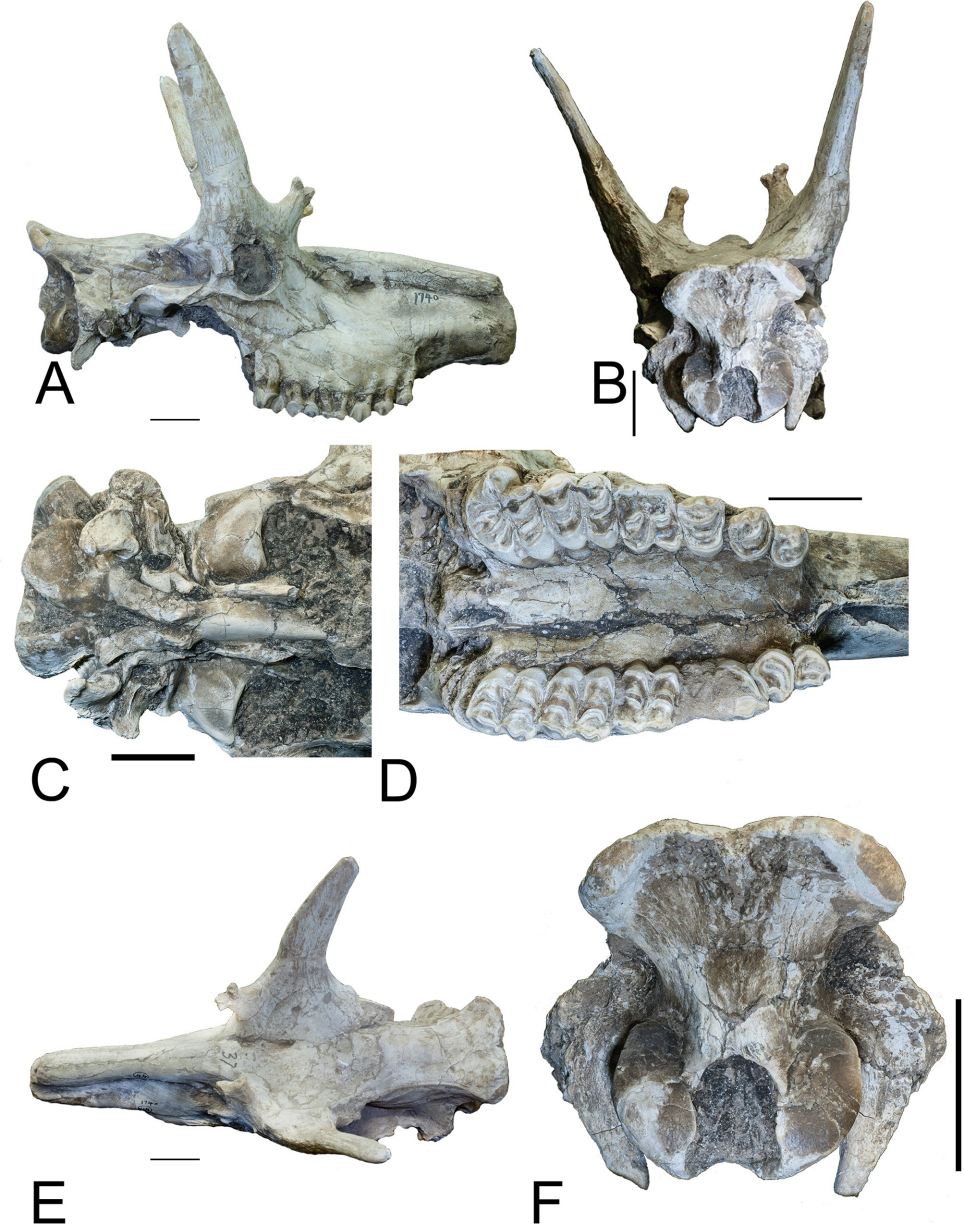
*Schansitherium tafeli* skull HMV 1740. (A) Right lateral aspect. (B) Occipital view. (C) The ventral braincase. (D) Palatal view of dentition (right M3 is abnormal). (E) Dorsal view. (F) Close view of occiput. Scale 50mm.

**Fig 2:**
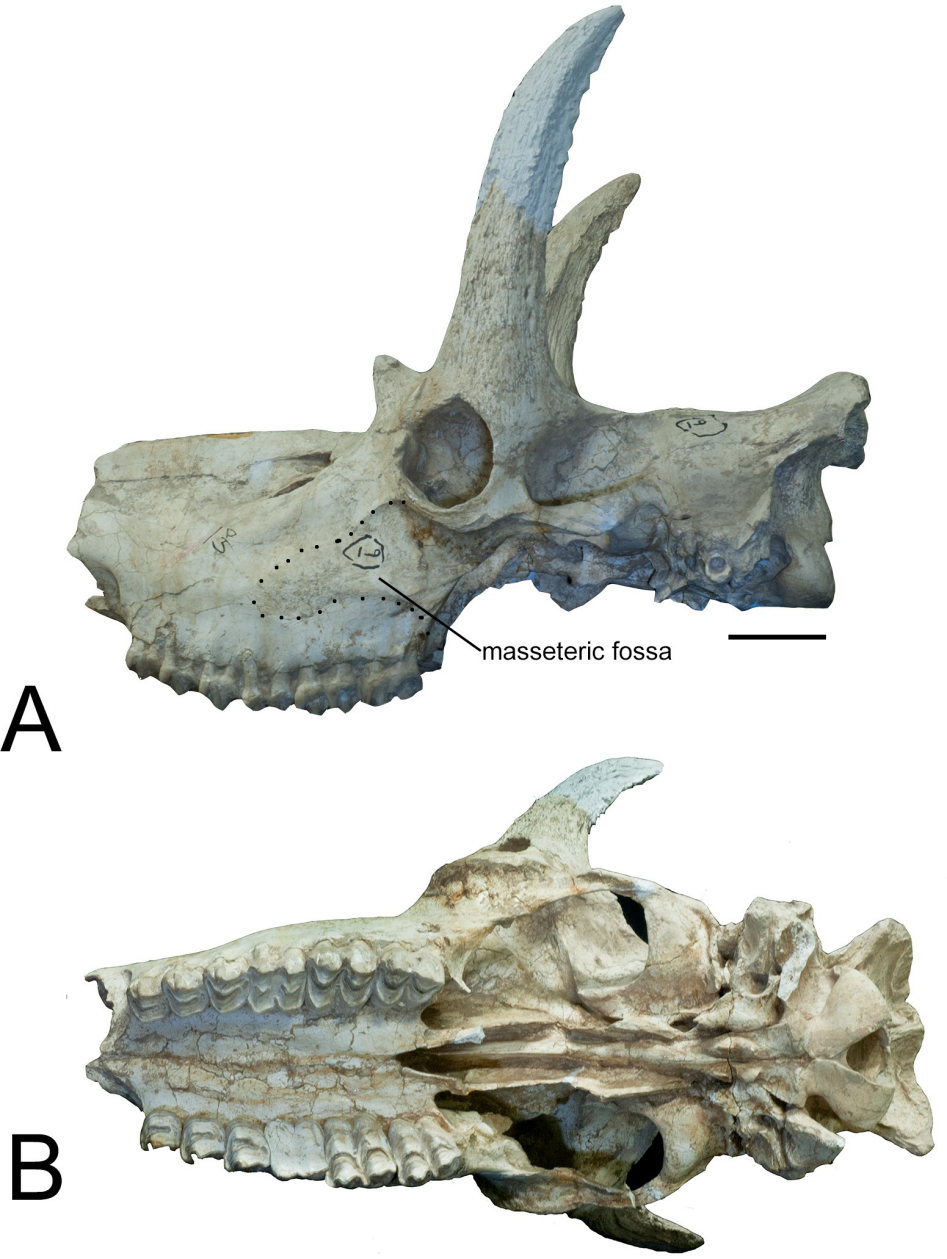
*Schansitherium tafeli* skull HMV 0945. (A) Left lateral aspect. (B) Ventral view. Scale 50mm.

**Fig 3:**
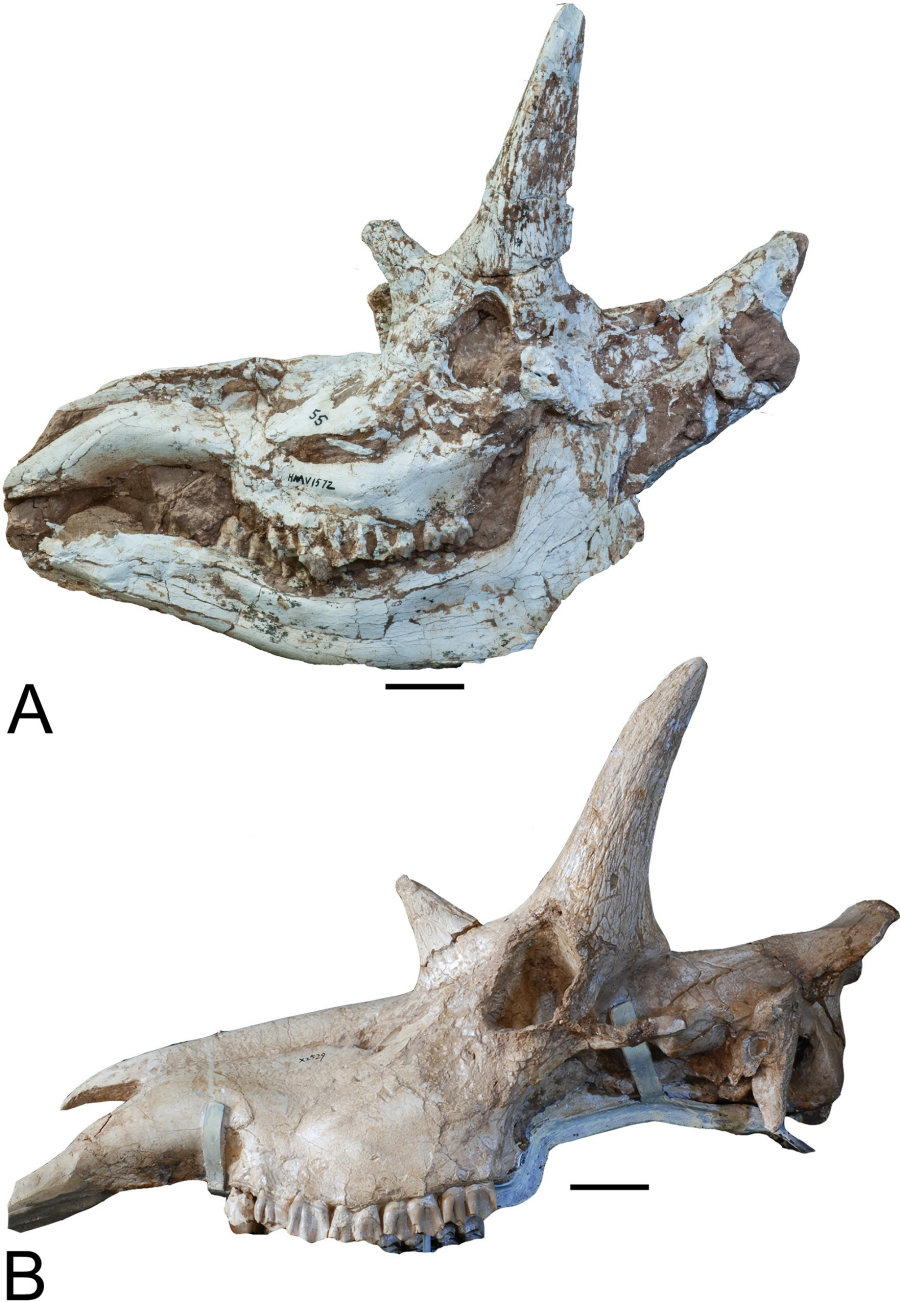
*Schansitherium tafeli* skulls. (A) HMV 1943 left lateral aspect. (B) HMN 1934 left lateral view. Scales 50mm.

**Fig 4:**
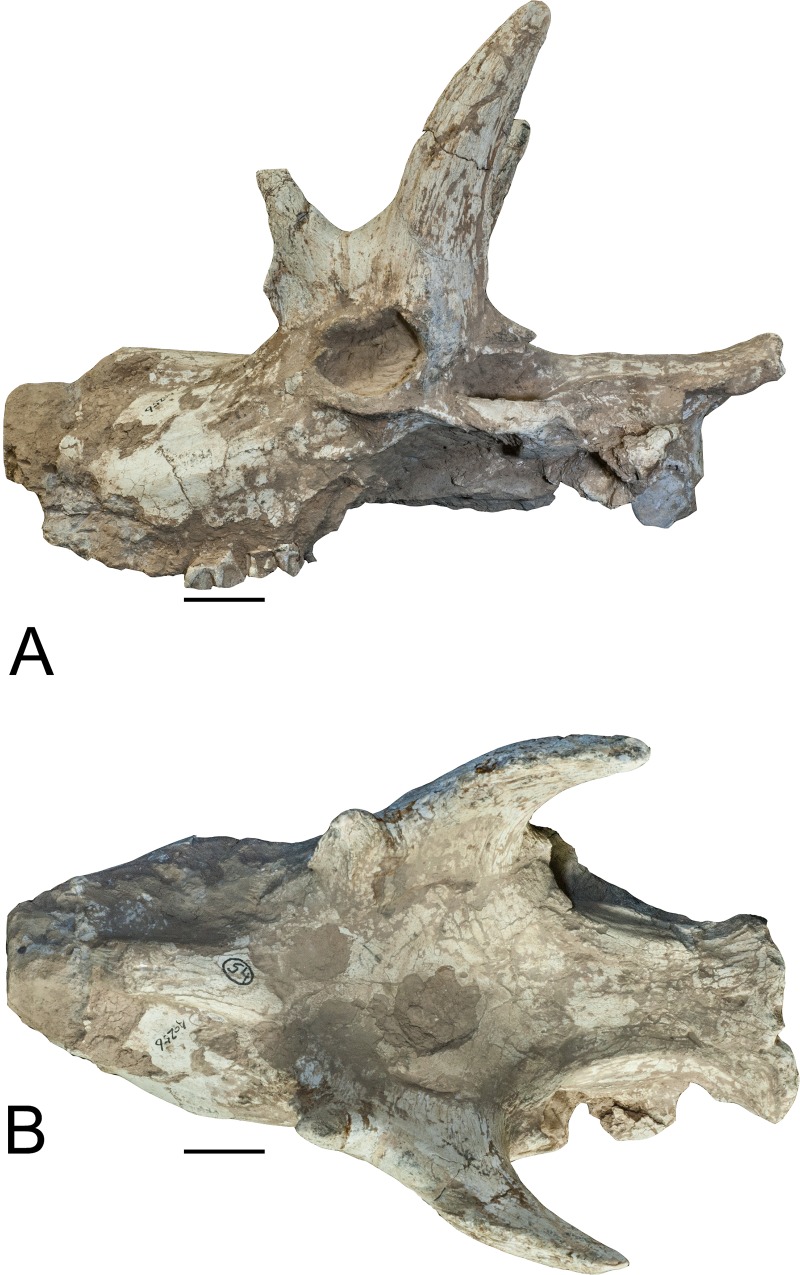
*Schansitherium tafeli*. (A) HMV 1931 left lateral aspect. (B) Dorsal view. Scale 50mm.

Four ossicones are present. The two pairs of ossicones share a common base on each side, which is positioned above the orbit. The base of the anterior pair is merged with the posterior pair, making the common base long and oval in cross section (Figs 1–4).

**Fig 5:**
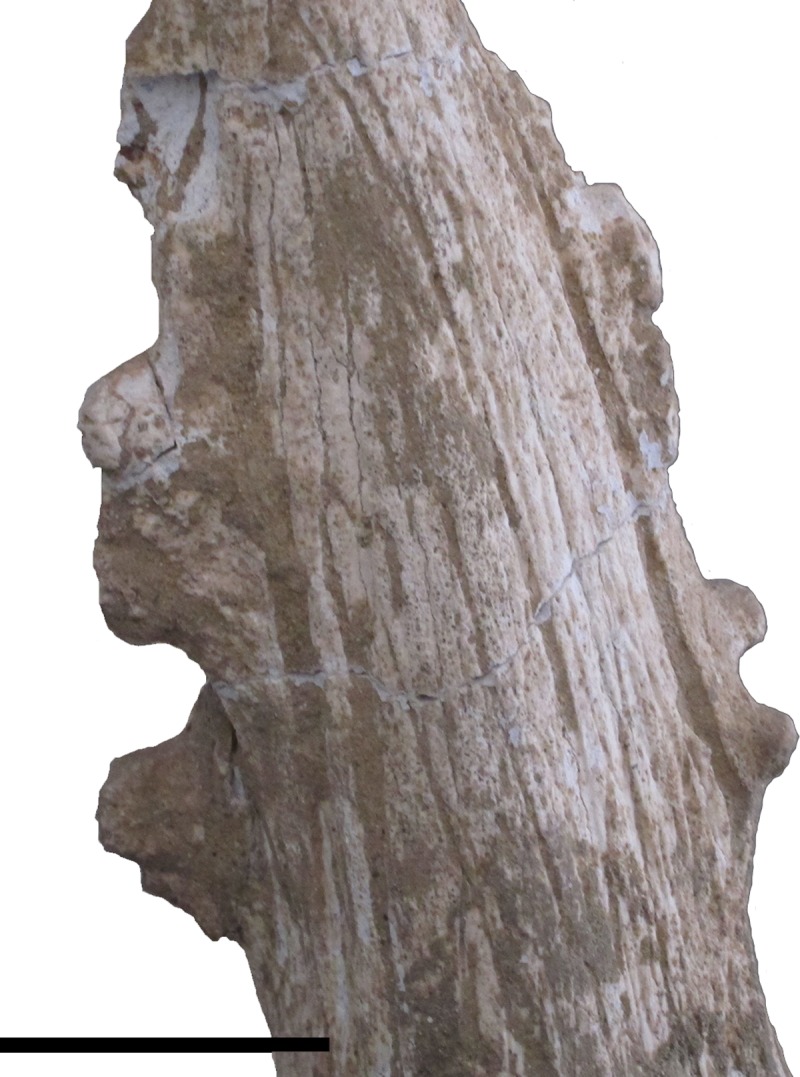
*Schansitherium tafeli*. (A) HMV 1321. Ossicone detail; right side lateral aspect. Scale 50mm.

**Fig 6:**
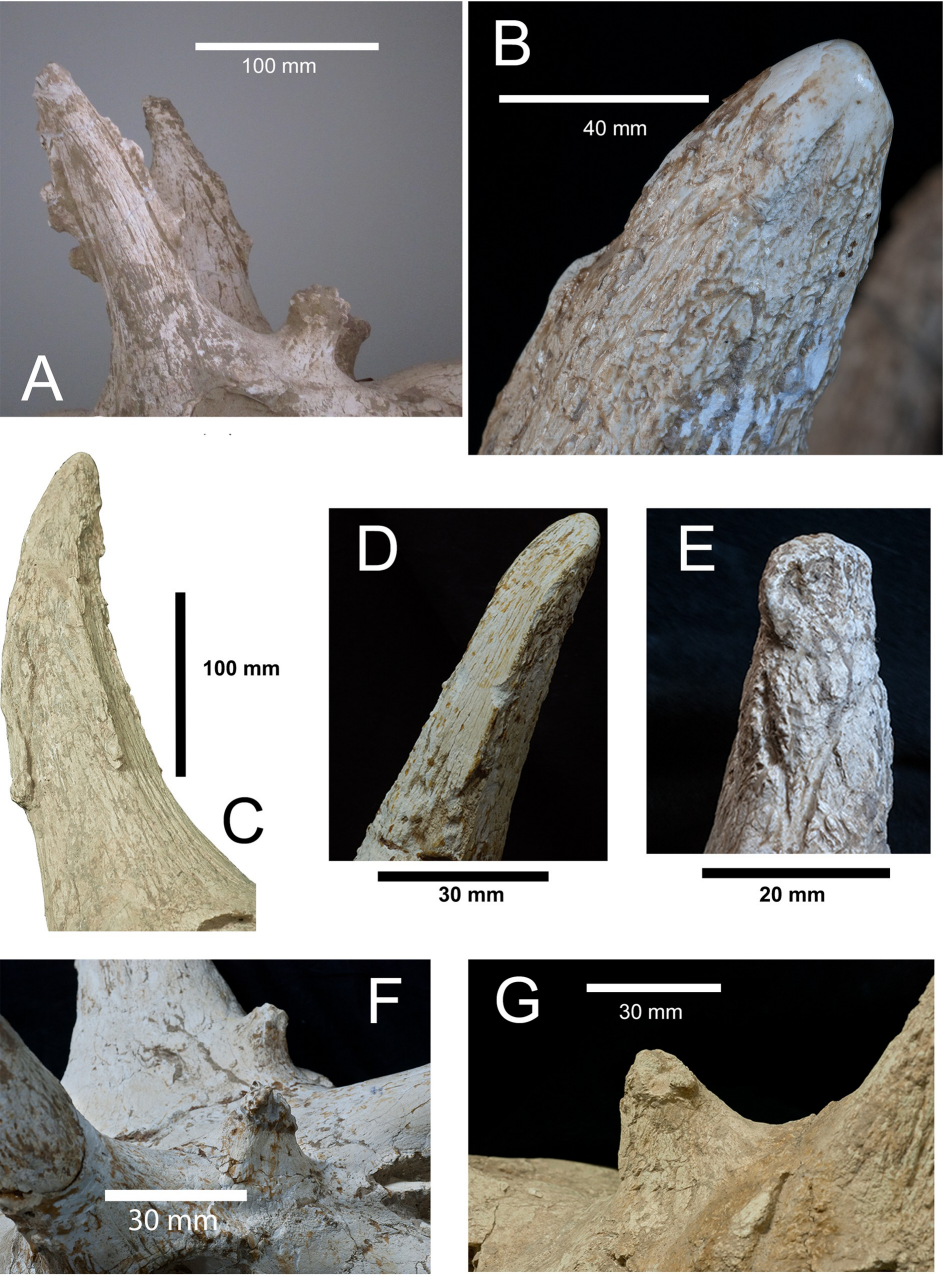
*Schansitherium tafeli*. (A) The two ossicones of HMV 1321 lateral aspect. (B) Medial aspect of HMV 1934. (C) Medial aspect of HMV 0945. (D) Apex of HMV 1932 medial aspect. (E) HMV 1934 apex. (F) Anterior ossicone of HMV 1932. (G) Medial aspect of ossicone HMV 1932.

The posterior ossicone is large and long, and is positioned above the orbit. The ossicone gently curves posteriorly, most notably at the distal end. The ossicone base is straight, compressed medio-laterally, and set slightly posterior to the orbit. The base laterally merges with the superior orbital rim. Thus, the rim is no longer distinct (Figs 1–4). There is considerable variation of the surface of these ossicones. The surficial grooving of the ossicone is irregular and forms long streaks separated by fine grooves. Certain specimens have this morphology (Fig 2). There are also secondary bone growth streaks descending from the apex forming four distinct ridges circumferentially in one specimen. Another specimen (Fig 5) has overgrown protrusions with lumpy appearance on the anterior and posterior ridges. The apex displays polish and planar wear facets on the anterolateral aspect of the ossicone (Fig 6B).

**Fig 7:**
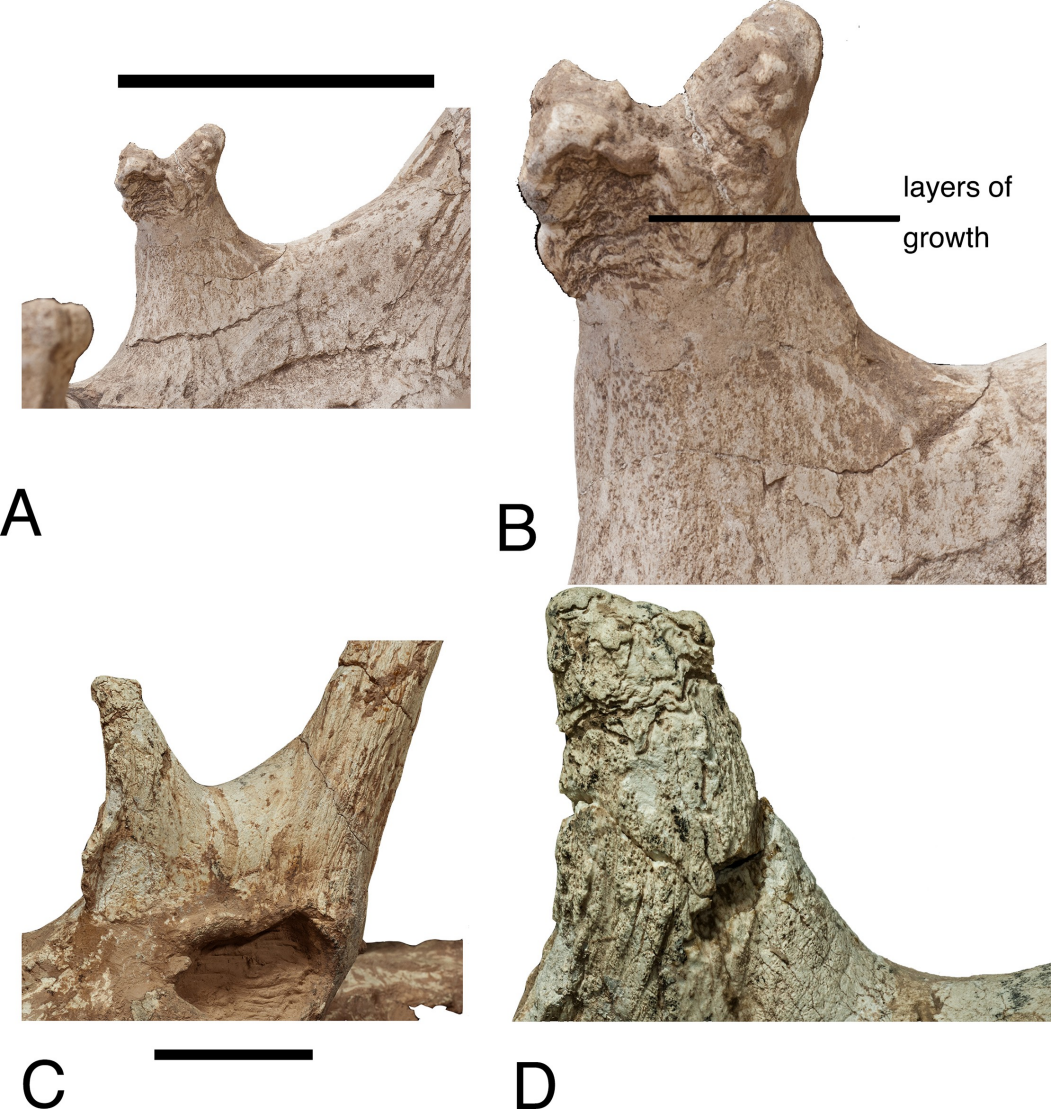
*Schansitherium tafeli*. (A) HMV 1740 medial aspect of anterior ossicone. (B) HMV 1740 close aspect of same specimen as Fig 7A showing layering. (C) HMV 1931 lateral aspect of orbit and ossicones. (D) HMV 1931 close image of 1931 showing layering. Scale 50mm.

The anterior ossicone is significantly shorter, and emerges from the base of the primary pair. The surface has the characteristic elongated ridges and streaks. At the apex, anterior ossicones have concentrations of secondary bone growth, which forms irregular and often multiple apices. In some specimens (interpreted as a variant morphology), the anterior ossicone forks at the apex, forming a “y” with one shaft directed anteriorly and the other directed posteriorly (Fig 7A and 7B). Presently, the nature of this variation is unclear. In the okapi, the apices vary a lot amongst individuals, and so far they are all considered to be a single species. The anterior subdivision is more irregular and wide, and the posterior portion is taller and rounder. In the majority of specimens, the anterior ossicone does not split at the apex, and is rounded with clustered secondary bone growth bumps. On one specimen, there is a wear surface at the anterior apex (Fig 7C and 7D). There are multiple semihorizontal layers of secondary bone growth, which we speculate to represent intermittent growth (Fig 7C and 7D). These horizontal layers have not been observed in any other taxon (Hou et al. 2014). In the giraffe, the secondary bone growth forms erratic patterns on the surface of the ossicone and skull, which is different from the horizontal layers of the *Schansitherium* anterior ossicones.

The paraoccipital process is directed medially in posterior view and in lateral view (Fig 1F). It ascends high to merge with the mastoid. The paraoccipital process is thin distally with a flattened posterior surface. The fossa for the occipitohyoid muscle is deep (Fig 1F). It is broad at the mastoid process area but then abruptly narrower above that point. The mastoid bone is massive in the posterior view and it extends substantially laterally and dorsally (Fig 1F). The nuchal ligament attachment is large, forms a deep and oval depression. The occipital tuberosity for the rectus capitis minor muscle is small. The occipital dorsal margin forms an indentation at the median plane. The center of the occipital forms a narrow ridge. Below this ridge and above the foramen magnum there is a medial oval fossa. There are no bony thickenings at the dorsal side of the foramen magnum. The occipital condyles are large and extend dorsally above the foramen magnum. In ventral view, they are separated by a median groove (Fig 1C). The occipital condyles tend to be approximated, meaning the median groove is extremely narrow. The mastoid in occipital view is well developed and extends dorsally to the nuchal crest.

**Fig 8:**
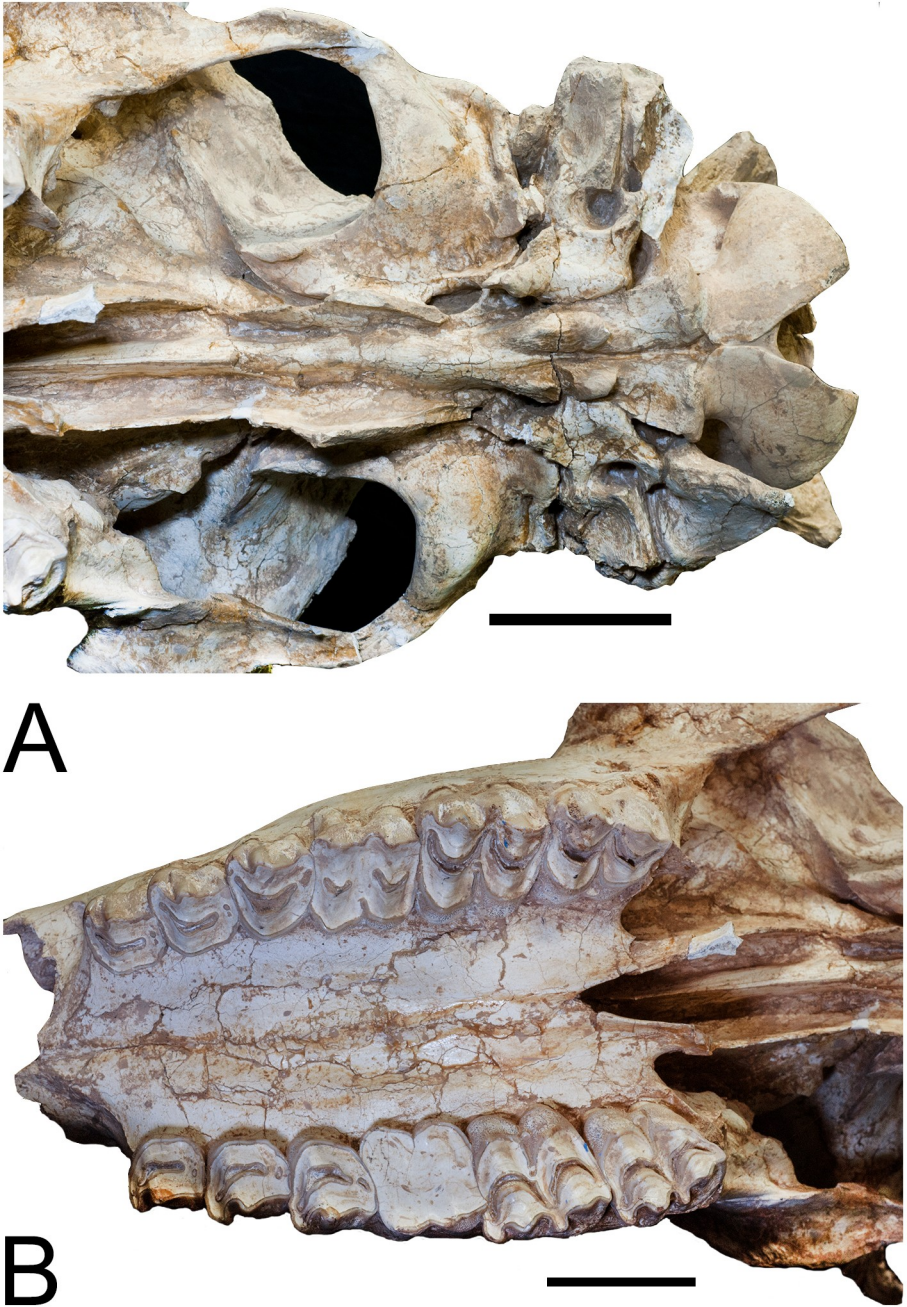
*Schansitherium tafeli* skull HMV 0945. (A) Basicranium. (B) Palate view. Scale 50mm.

The anterior basioccipital tuberosities are oval and well-developed (Fig 8A). The posterior basioccipital tuberosities are broad and more flattened, but are not robust. The tuberosities are separated from each other by a median wide space. The anterior and posterior tuberosities are connected. There is a sharp ridge, which comprises the ventral edge of the basioccipital bone. The bulla is broad squared in ventral view forming a flat surface. The most ventral central area of the ectotympanic forms a small additional dome. Anterior lateral to the bulla is an elongation where the Eustachian tube is situated (Fig 8A). The external auditory meatus has a deep fossa anteriorly and a ridge on the posterior edge.

The orbital rim edge is covered dorsally by the ossicone. The zygomatic is flat and broad. The masseteric fossa is of medium size and the orbit is set above the third molar. At the level of M1 posterior margin, the masseteric fossa has a triangular rough area. Muscle fibers were strongly and differently attached there from the rest of the masseteric fossa (Fig 2A). The ethmoidal fissure is small and open and an ethmoid plate subdivides the o pening into two sub-equal areas (Figs 1–4). The zygomatic edge for zygomandibularis is of medium robusticity. Posterior to M3 there is no bony extension and the third molar is at the very edge of the maxilla. There is a bony spike on the posterior bony surface of M3 (Fig 8B).

**Fig 9:**
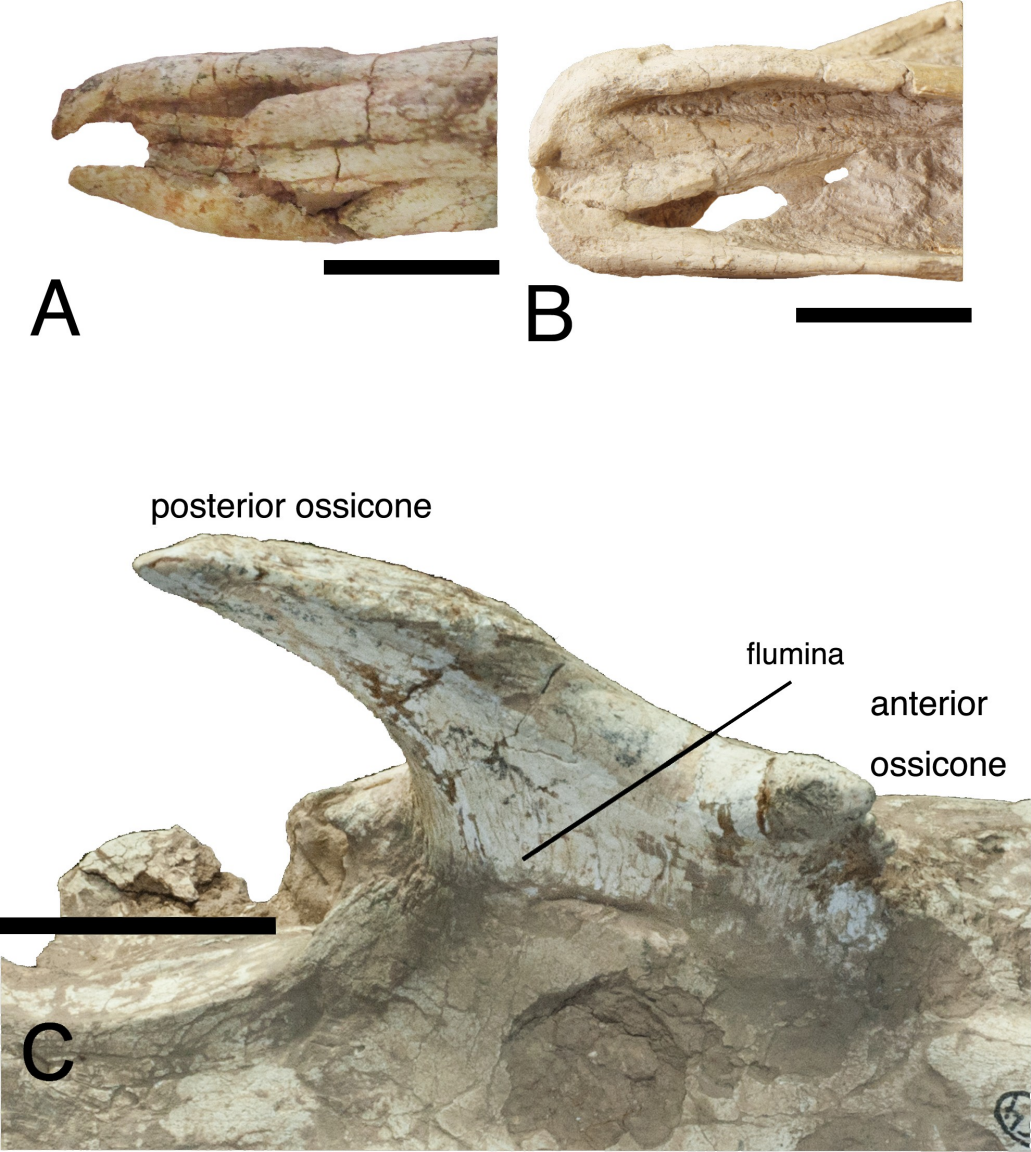
Premaxillae and ossicone. (A) Premaxilla HMV 1416, *Schansitherium tafeli* from Gansu. (B) Premaxilla *Samotherium boissieri* from Samos NHMUK 1415. (C) *Schansitherium tafeli* HMV 1931 left ossicone. Scale 50mm.

The premaxilla is overall pointed but turns abruptly inwards at the very distal end (Fig 9A). The diastema is short for a giraffid (Fig 3A). Colbert has quantified the diastema propotions of *Giraffa* and *Okapia* and extinct giraffids (*Samotherium* sp., *Giraffokeryx*, and *Palaeotragus*). Using the length between the anterior premolar and the tips of the premaxillaries (or the incisors in the mandible) to indicate the diastema, the ratio of the diastema length to premolar-molar length (Premolar-molar length = 100) are calculated as 112–153 for the giraffids he studied [26]. The *Schansitherium* diastema in our study has a ratio of approximately 75–85 (Fig 3A). The upper premolars are reduced in size and the styles are small and simple (Fig 8B). The P2 has a strong anterior wall but the parastyle is small (Fig 8B). The premolars and the molars are typical giraffid with medium-sized styles and ribs. There are no lingual cingula. There are basal pillars in some (Fig 8B). The choanae (median palatine indentation) are set at the level of the middle M3. The indentation forms a narrow V (Fig 8B).

The calvaria is typical giraffid with two strongly formed temporal ridges. The supraorbital foramina are small and are set on the medal surface of the ossicone. Near the ossicone and the supraorbital foramen, there are deep grooves in the skulls of giraffids, which appear to connect with the supraorbital foramen. We propose the name flumina for them, meaning rivers in Latin, as they have not been previously described previously. The flumina are variable, as some specimens of *Schansitherium* have very few while others have more. Other specimens have an extended set of flumina, which ascend onto the ossicone (Fig 9C). The boss for the posterior ossicone is positioned laterally, at the edge of the orbit. The nasals in lateral view are slightly bowed. In dorsal view they are wide.

### Third cervical vertebra

**Fig 10:**
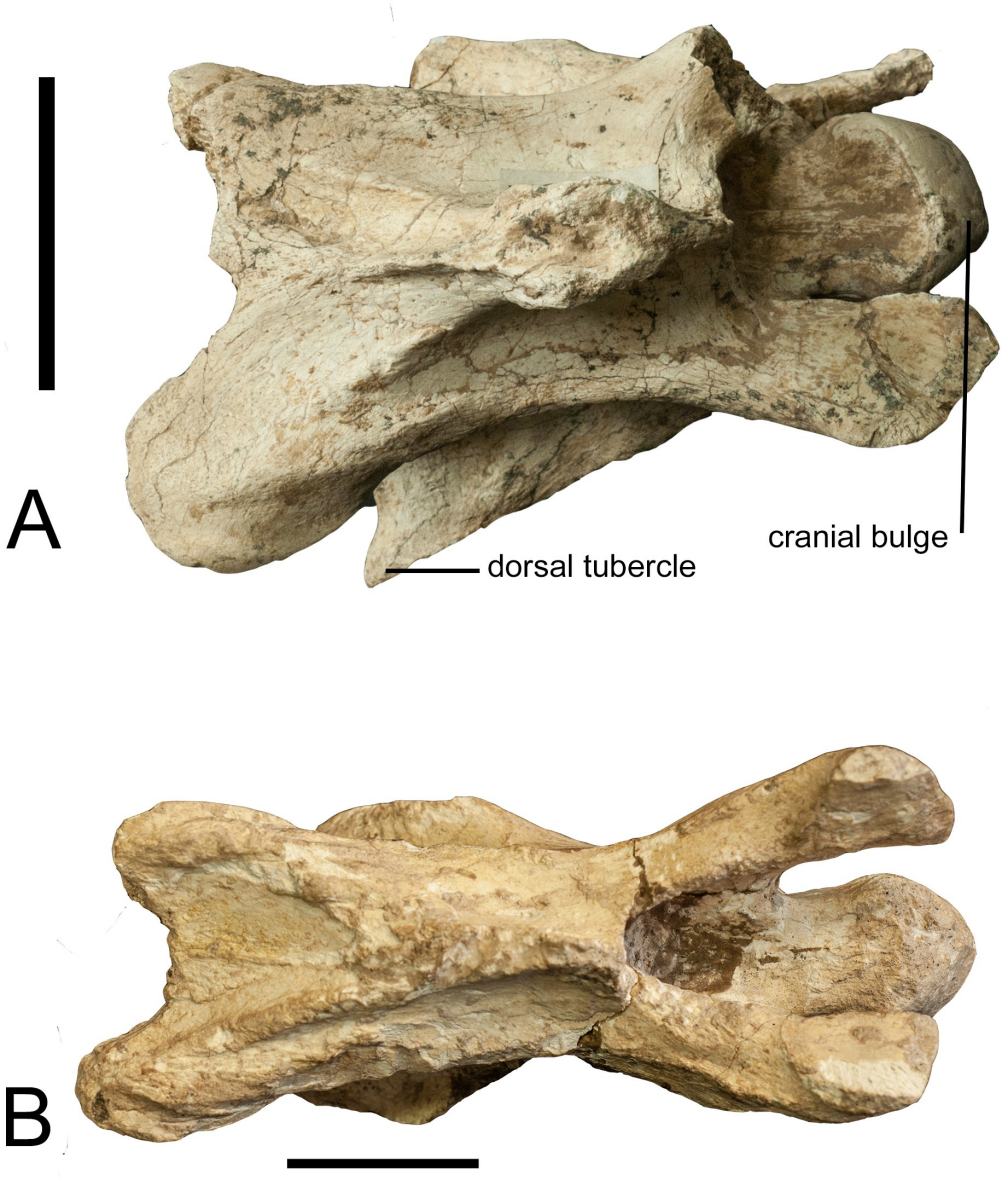
Comparison of cervical vertebrae. (A) Dorsal view of the third cervical vertebra of *Schansitherium tafeli* HMV 1988 from Gansu. (B) Dorsal view of third vertebra of *Samotherium boissieri* NHMUK 4250 from Samos. Scale 50mm.

**Fig 11:**
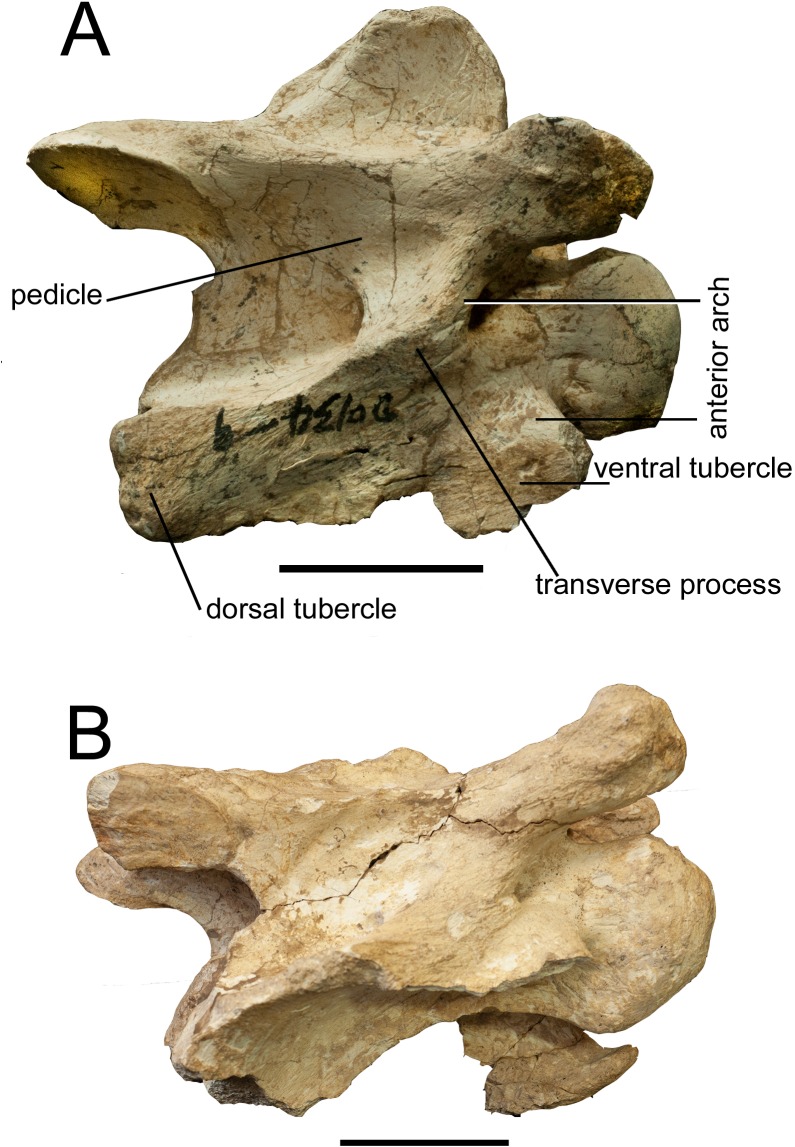
Comparison of cervical vertebrae. (A) Lateral view of the third cervical vertebra of *Schansitherium tafeli* HMV 1988 from Gansu. (B) Lateral view of third vertebra of *Samotherium boissieri* NHMUK 4250. Scale 50mm.

The vertebra was evaluated and the terminology we use is based on the following studies: Danowitz and Solounias [24]; Danowitz et al. [5, 25]. Table 3 summarizes the morphology. The vertebra is short (Fig 10A). The spinous process is bifid with a flattened apex, and is directed slightly cranially (Fig 10A). Three distinct ridges radiate from the caudal aspect of the spinous process onto the dorsal lamina. The cranial bulge is elongated and flat, and it separates the cranial articular facet from the dorsal lamina. The caudal portion of the dorsal lamina is notched between the caudal articular processes. The articular facet is semilunar in shape from dorsal view. The cranial articular process is elongated but flattened. In lateral view, the transverse process presents as a raised, thin, elongated ridge that extends between the openings of the transverse foramen towards the caudal vertebral body (Fig 11A). There is no distinct dorsal tubercle visible (Fig 11A). The caudal opening of the transverse foramen is at the middle of the vertebral body. The ventral tubercle is directed cranially. The anterior arch is interrupted, so that the base of the cranial articular process is positioned caudally, and there is no continuous ridge connecting the cranial article facet with the ventral tubercle. The cranial bulge is domed and spherical. In lateral view, the caudal aspect of the vertebra is notably larger than the cranial part. In ventral view, the cranial end of the vertebra is significantly narrower than the caudal end. The ventral ridge is prominent and is continuous longitudinally on the vertebral body (Figs 10 and 11). The region caudal to the transverse foramen is elongated and the region anterior to the transverse foramen is short. The pedicle between the foramina transversaria is vertical.

**Table 3:** Evaluation of cervical elongation.

	Character	*Schansitherium tafeli*	*Samotherium boissieri*
General elongation	shape of cranial articular facet	elongated	elongated
	directionality of shaft of ventral tubercle	elongated	elongated
	height of the intertubercular plate	not elongated	elongated
	strength of ventral ridge	not elongated	elongated
Cranial elongation	shape of cranial bulge	not elongated	elongated
	prominence of ventral extension	not elongated	elongated
	ventral tubercle in relation to cranial bulge	elongated	elongated
	connection of cranial articular facet with lamina	elongated	elongated
	continuity of anterior arch	elongated	elongated
Caudal elongation	height of spinous process	not elongated	elongated
	highest point of spinous process in relation to the foramen transversarium	intermediate	intermediate
	position of spinous process thickness	intermediate	intermediate
	presence of laminar ridges	not elongated	not elongated
	dorsal tubercles in relation to the vertebral body	not elongated	not elongated
	position of caudal articular facet	not elongated	not elongated
	ventral notch	not elongated	not elongated

### Metacarpal

**Fig 12:**
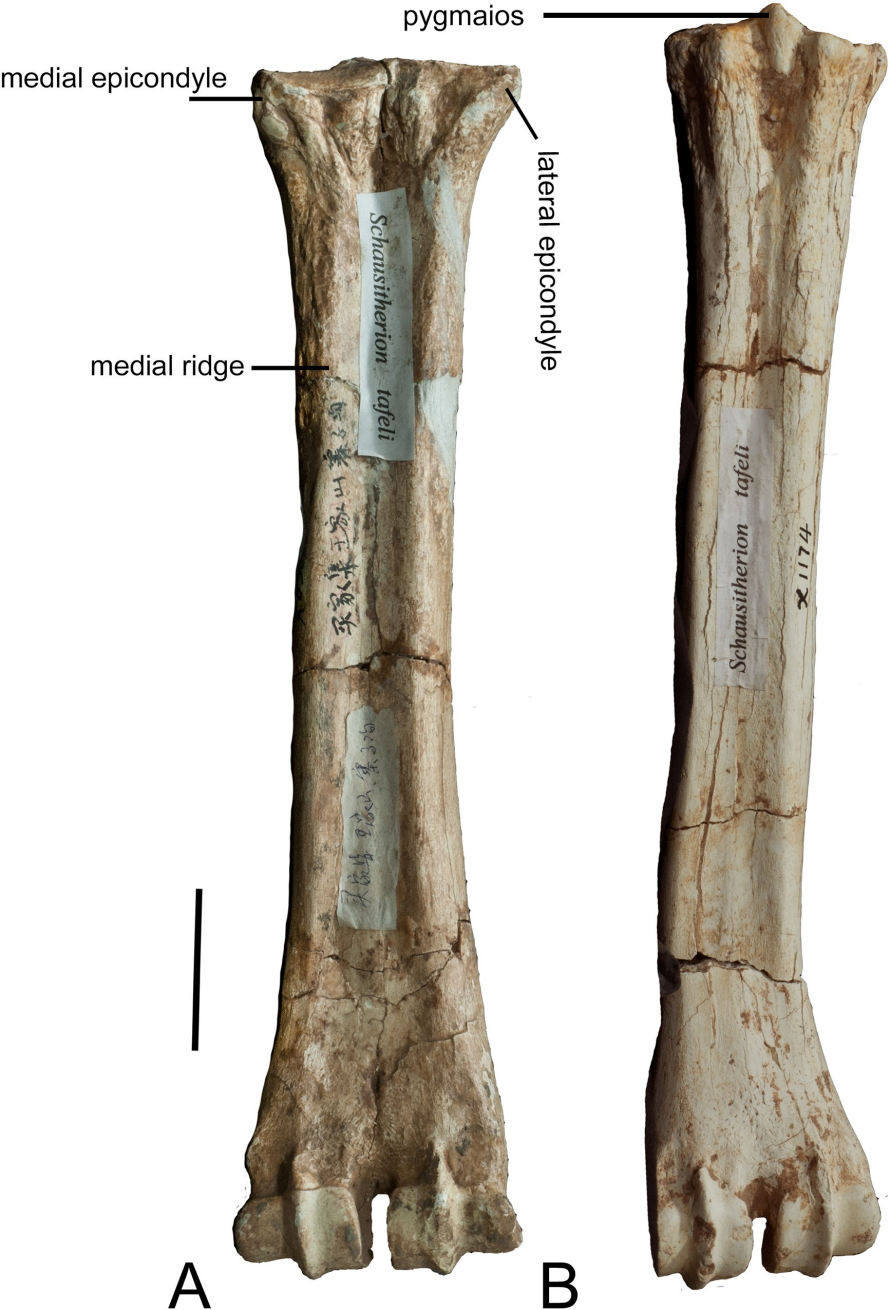
*Schansitherium tafeli*. (A) Ventral view of right metacarpal HMV 1951. (B) Ventral view of right metatarsal HMV 1986. Scale 50mm.

The metacarpals were evaluated after Rios et al. [6]. The epicondyles are similar in size and morphology. The lateral epicondyle is slightly wider and fuller. Grooves separate the lateral epicondyle into three distinct heads. The medial thickening continues onto the lateral ridge. Lateral to the ridge there is an elongated facet. The proximal articular surface extends slightly onto the ventral surface of the lateral epicondyle. An obliquely oriented, wide groove separates the lateral epicondyle into a laterally flaring head. The medial epicondyle is slightly flatter. One groove separates the medial epicondyle into two distinct heads (thickenings). The groove is obliquely oriented. There is a deep narrow groove that separates the epicondyles centrally. The medial ridge emanates from the lateral thickening. The medial ridge is sharper and thinner, and it flattens towards the distal shaft. The lateral ridge is rounder and thicker, and also flattens distally. The central trough is deep throughout the proximal and mid-shaft, and it flattens towards the distal condyles. The pyramidal rise is faint. The distal shaft has a notable flare medially and laterally, creating a spatula-like shape. The keels of the distal condyles extend ventrally onto the distal shaft (Fig 12A).

### Metatarsal

The medial and lateral epicondyles are similar in size and morphology. The lateral epicondyle has a rounded surface, and it is oriented longitudinally. It is separated into a dorsal head and a ventral head by a deep longitudinal groove. The ventral head is oriented longitudinally with the long axis of the main shaft, and the dorsal head flares outward. The medial epicondyle has a slightly flatter surface, and is not divided into multiple heads. The medial epicondyle is directed medially. The lateral and medial epicondyles are separated centrally by a narrow, deep groove that continues onto the central trough. The medial and lateral epicondyles are continuous distally with the medial and lateral ridges, respectively. The pygmaios [6] is oriented closer to the medial epicondyle. There is an elongated, oval bony protrusion at the proximal medial shaft, which originates at the level of the proximal articular surface. This protrusion is directed obliquely towards the ventral surface, and is present at the proximal-most shaft. The medial and lateral ridges continue to the distal shaft, where they flatten proximal to the condyles. The central trough is intermediate in depth. There is a significant pyramidal rise present throughout the majority of the shaft. The distal shaft has a notable flare laterally. The keels of the distal condyle continue slightly onto the ventral shaft (Fig 12B).

## Brief description of *Samotherium boissieri*

Type species: *Samotherium boissieri* Major, 1888

Type locality: Samos

Holotype: A skull with damaged basicranium and partially broken ossicones. The skull is crushed towards the middle. The occipital is damaged in the specimen NHMUK 4215.

The holotype has been described by Geraads [16], Hamilton [2], and Kostopoulos [23]. The new skulls from Gansu clearly shows all the features of *Samotherium boissieri* from Samos, and these specimens are listed under the materials section. Some of these features shared between the new skulls from Gansu and the previously diagnosed specimens NHMUK 4215 and NHMUK 4216 include the shape of the occipital, the dentition, the details of the Basicranium, the position and shape of the ossicones, the masseteric fossa size, and the details of the lacrimal bone.

### Cranial

**Fig 13:**
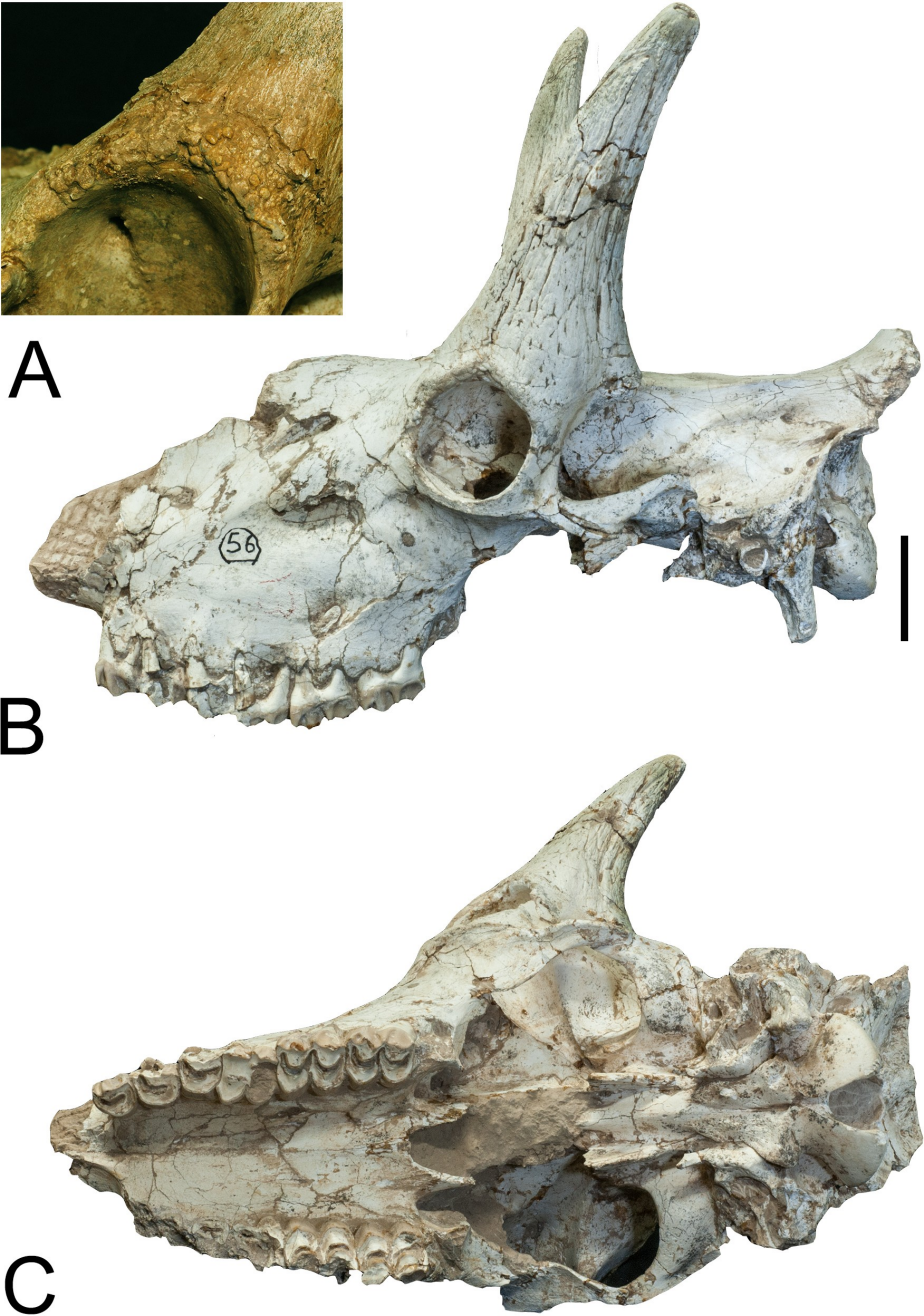
*Samotherium boissieri*. (A) IVPP V20167 base of left ossicone. (B) Lateral aspect of skull IVPP V20271. (C) Ventral view of skull IVPP V20271. Scale 50mm.

**Fig 14:**
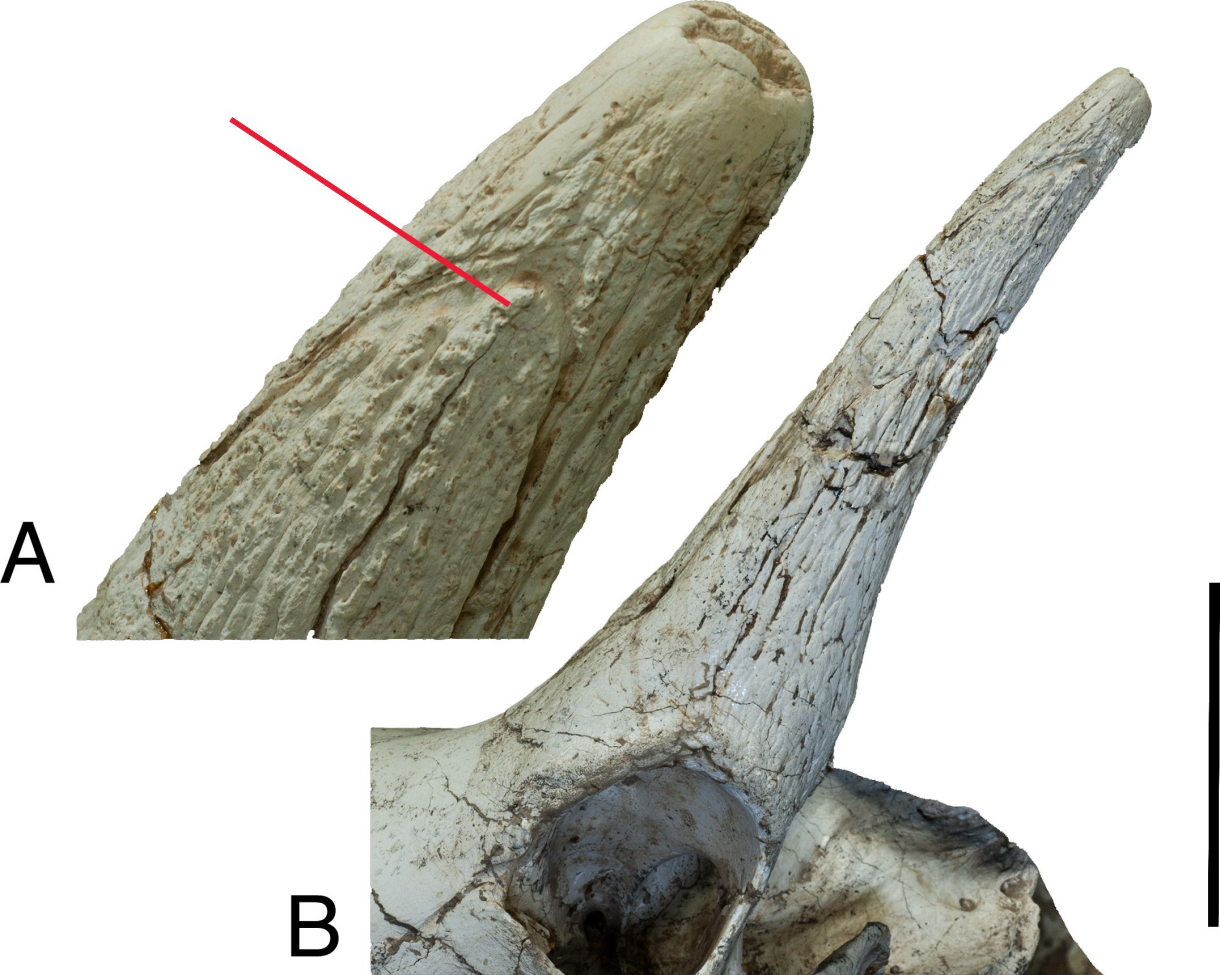
*Samotherium boissieri*. (A) IVPP V20271 ossicone close-up. Red line points to para-apex. (B) Another view of IVPP V20271 ossicone. Scale 50mm.

**Fig 15:**
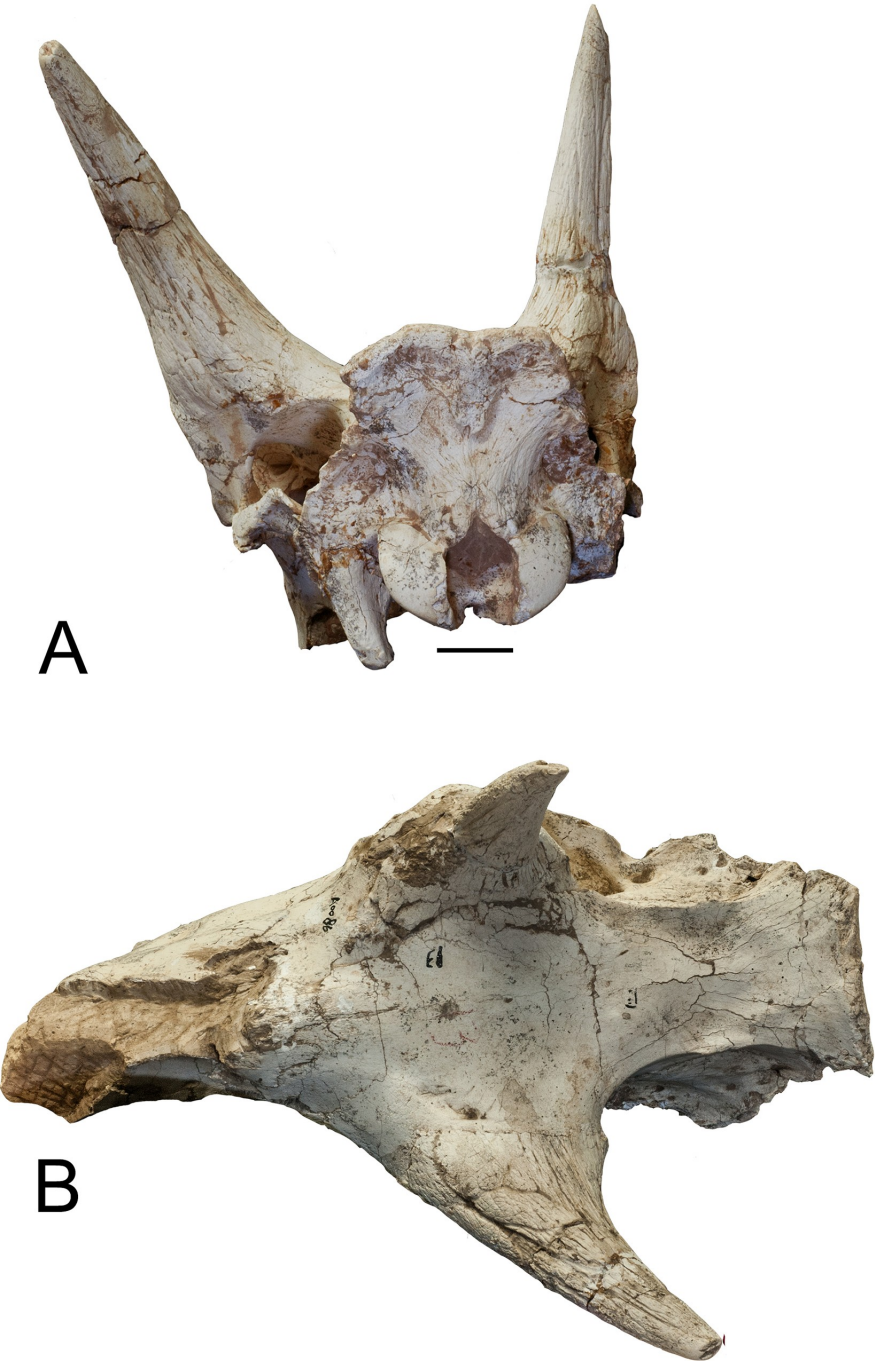
*Samotherium boissieri*. (A) Occipital aspect of skull HMV IVPP V20271. (B) Dorsal view of skull. Scale 50mm.

**Fig 16:**
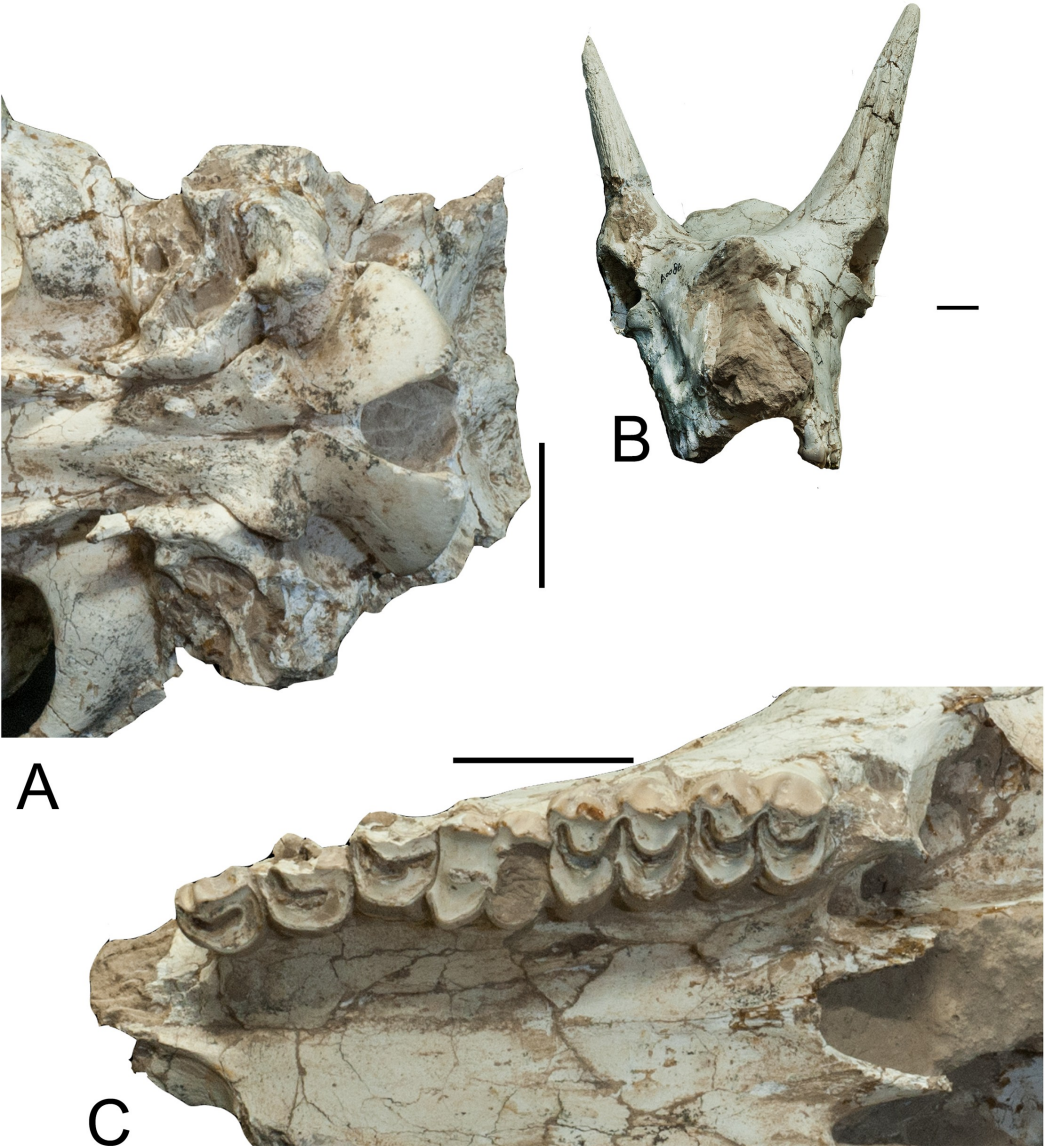
*Samotherium boissieri*. (A) Ventral aspect of braincase HMV IVPP V20271. (B) Anterior aspect of skull. (C) Upper dentition. Scale 50mm.

We provide a new skull of *Samotherium boissieri* which is much more complete and better preserved compared to the skulls from Samos in European museums. Two cylindrical spike-like ossicones are present. The ossicone base is oval and the ossicones are positioned vertically superior to the orbit (Fig 13). The base of the ossicone is about twice the size of the orbit. The surficial grooving of the ossicone is minimal, irregular and forms long streaks separated by shallow fine grooves. The apex of the ossicone has apical polish and planar wear facets. Near the apex of the ossicone, there is convergence of the grooving, forming a point like another apex. We find this to be widespread among Giraffidae, and we give it the new term para-apex (Fig 14). This is the location where palaeomerycid ossicone bends abruptly, which is why it is deserving of its own term [27]. On the streaks in some specimens there are overgrown protrusions with lumpy appearances. The secondary bone growth appears to have formed on the surface of the frontal bone under the ossicone. Therefore, the ossicone overrides the secondary bone growth (Fig 13A). In lateral view the face is deep compared to the braincase. The nasals are domed. The masseteric fossa is large. The postorbital bar is slightly twisted. The premolars are small and the styles are reduced. The border of the posterior palatine bone forms a U-shape. The brain case is long and the temporal ridges are faint. There is a characteristic extension and turning dorsally of the occiput caudally in lateral view, which is also seen in *Decennatherium*. The mastoid is massive and it extends substantially dorsally merging with the occipital crest (Fig 15). The anterior and posterior basioccipital tuberosities are connected laterally by a notably sharp ridge. The ridge is the edge of the basioccipital. The bulla is compressed in ventral view (Fig 16A). The external auditory meatus has a deep fossa anteriorly and a ridge on the posterior edge. The glenoid fossa is domed and the post glenoid process continues laterally with a ridge. The masseteric fossa is medium in development. The diastema is long. The supraorbital foramina are notably small and are positioned on the medal surface of the ossicone. There are small flumina associated with the supraorbital foramina and with the groove anterior to the foramina. The flumina are medial to the foramina (Fig 15B). The nasal-frontal suture is more arched.

### Third cervical vertebra

In dorsal view, the spinous process is situated cranially on the lamina, towards the base of the cranial articular processes (Fig 10B). The spinous process is wide cranio-caudally, and U-shaped in lateral view. The spinous process extends the majority of the length of the lamina. There are three distinct ridges that emanate from the caudal portion of the spinous process; the central ridge is thinner and fainter than the outer ridges. The cranial articular facets are oriented dorso-medially and they are oval shaped. The cranial articular process is elongated and rounded. In lateral view, the transverse process forms a rounded ridge that connects distally to the dorsal tubercle. The ventral tubercle is well preserved; it is oriented cranially and has a thickening at the ventral-most aspect. The cranial portion of the ventral tubercle is hooked. The anterior arch is interrupted in lateral view. The cranial bulge is spherical. In ventral view, there is a distinct ventral extension of the cranial bulge. The ventral ridge is tall and prominent, and it extends the entire length of the ventral vertebral body (Fig 11B).

### Metacarpal

**Fig 17:**
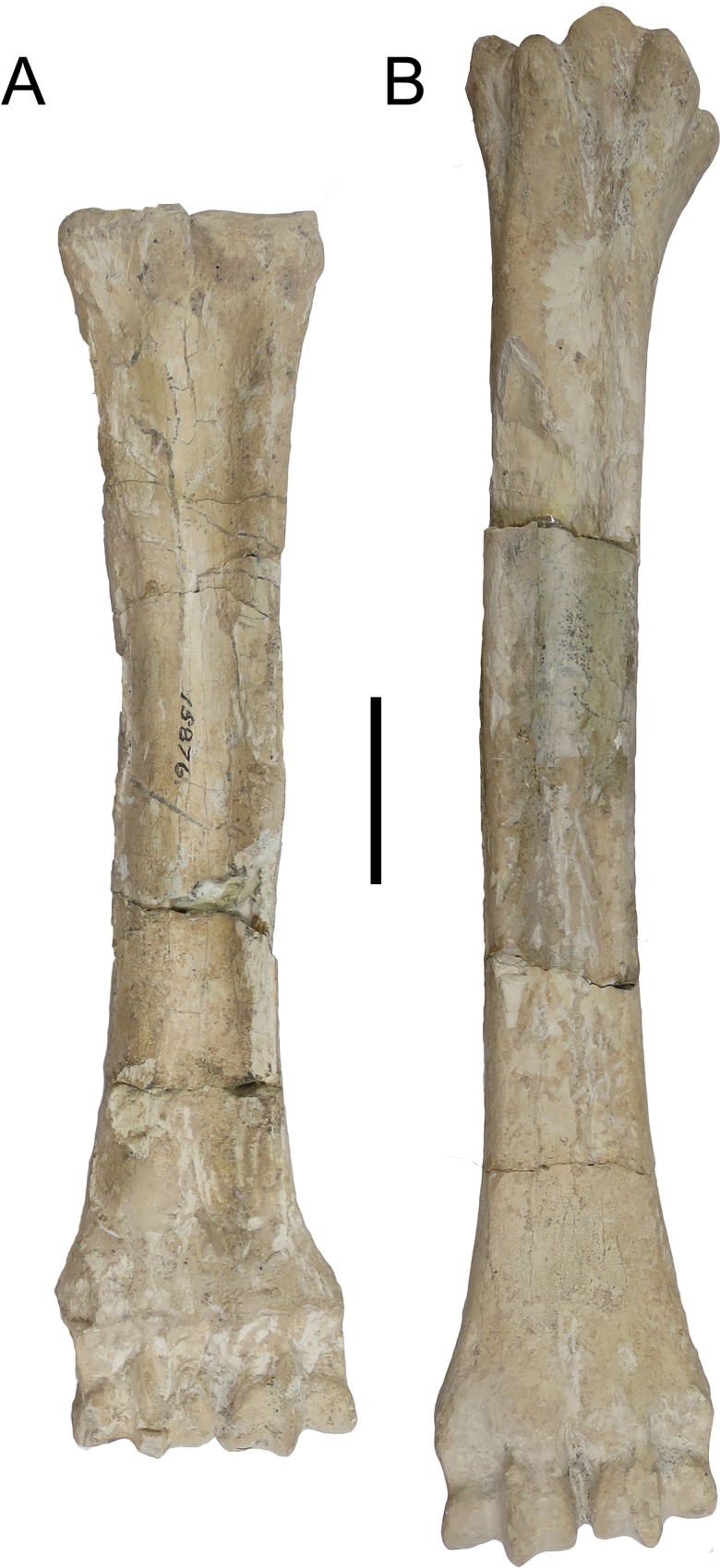
*Samotherium boissieri* metapodials from Samos. (A) Right metacarpal AMNH 15867. (B) Left metatarsal AMNH 15867. Scale 50mm.

The epicondyles are similar in size and morphology. A thickening continues onto the lateral ridge. The proximal articular surface extends slightly onto the ventral surface of the lateral epicondyle. The medial ridge emanates from the medial epicondyle. The medial ridge is sharper and thinner, and it flattens towards the distal shaft. The lateral ridge is rounder and thicker. The central trough is medium in depth. The pyramidal rise is faint. The distal shaft has a notable boxy shape medially and laterally. The keels of the distal condyles do not extend ventrally onto the distal shaft (Fig 17A).

### Metatarsal

The medial and lateral epicondyles are similar in size and morphology. The lateral epicondyle has a rounded surface, and it is oriented longitudinally. It is separated into a dorsal head and a ventral head by a deep longitudinal groove. The ventral head is oriented longitudinally with the long axis of the main shaft, and the dorsal head flares outward. The medial epicondyle has a rounded surface, and is divided into multiple heads. The medial epicondyle is directed medially. The lateral and medial epicondyles are separated centrally by a narrow, deep groove that continues onto the central trough. The medial and lateral epicondyles are continuous distally with the medial and lateral ridges, respectively. The pygmaios is large and oriented closer to the medial epicondyle. There is an elongated, oval bony protrusion at the proximal medial shaft, which originates at the level of the proximal articular surface. This protrusion is directed obliquely towards the ventral surface, and is present at the proximal-most shaft. The medial and lateral ridges continue to the distal shaft, where they flatten proximal to the condyles. The central trough is shallow in depth. There is a significant long pyramidal rise present throughout the majority of the shaft. The distal shaft has a slight flare laterally. The keels of the distal condyle do not continue slightly onto the ventral shaft (Fig 17B).

## Discussion

### Comparison of *Schansitherium* and *Samotherium*

While *Schansitherium tafeli* and *Samotherium boissieri* are similar taxa in cranial and post-cranial morphology (see introduction), the greatest difference between the two genera is in the ossicones. The posterior pair of ossicones is very similar to that of *Samotherium*; however the ossicones of *Schansitherium* occasionally possess bumps on both sides indicative of localized secondary bone growth. The ossicones also have deeper grooves than in *Samotherium*. The anterior pair of ossicones of *Schansitherium* is unique among giraffids. Its apex divides to sometimes two or three branches. Below these divisions, multiple horizontal layers of growth are visible. At the base, the anterior and posterior ossicones on each side converge to a common base. The formation of a saddle between the anterior and posterior ossicone is unique to *Schansitherium*. In giraffids that exhibit two pairs of ossicones, including *Giraffokeryx*, *Bramatherium*, *Decennatherium*, and *Sivatherium*, the anterior ossicones are separate from the posterior ones [10, 17].

*Schansitherium* and *Samotherium* also differ in the premaxillary shape. In *Schansitherium*, the premaxilla is of the intermediate type between squared and pointed, indicative of a mixed diet. Accordingly, mesowear studies of the dentitions of *Schansitherium tafeli* also indicate that this taxon had a browsing to mixed feeding diet [19]. In *Samotherium*, the premaxilla is squared, which is a more specialized condition indicative of grazing [28]. The mesowear analysis of *Samotherium boissieri*, however, indicated a more mixed feeding diet in localities in both China and Greece [19]. Recent studies have questioned the premaxillary shapes, suggesting that shape does not always specify diet, so future studies of *Schansitherium* specimens need to be reinterpreted [29]. In our interpretation, it visually appears to have been a mixed feeder.

The third cervical vertebra of *Schansitherium* demonstrates that this giraffid had a shorter neck than that of *Samotherium*. In giraffid evolution, neck elongation occurs in three stages; general vertebral elongation precedes the start of the Giraffidae, cranial elongation occurs around the start of the samothere lineage, and caudal elongation allows for the extreme elongation of the modern giraffe [5]. Overall, the third cervical vertebra of *Samotherium boissieri* and *Schansitherium tafeli* are relatively similar, and they differ in five of the total sixteen vertebral elongation characters that were scored. *Samotherium boissieri* exhibits more features of neck elongation when compared to *Schansitherium tafeli*; *Samotherium boissieri* exhibits a greater degree of cranial, caudal, and general elongation (Table 3). Elongation of the neck is a major adaptive change seen in several lineages in Bovidae (*Gazella dama* shorter neck and *Litocranius walleri* a longer neck) [30]. Among the Giraffidae, *Canthumeryx sirtensis*, as an early taxon, displays a relatively long neck. *Giraffa camelopardalis* and *Bohlinia attica* possess the longest necks in the family. The *Samotherium* neck exhibits an intermediate stage of elongation [25]. The elongation differences imply different fighting and feeding strategies from the shorter necked taxa to the longer [31].

While *Samotherium* is found in both southern Europe and northern China, *Schansitherium* has only been found in northern China. The absence of *Schansitherium* from southern Europe is interesting, but may be related to some sort of environmental differences that prevented the species from thriving there.

### Evolutionary relationships

*Schansitherium tafeli* has two ossicone pairs, a feature shared with *Giraffokeryx* and the Sivatheriinae, which could represent parallel evolution. According to the cladogram by Rios et al., *Schansitherium* is placed between two species of *Samotherium* [17]. This implies a very close relationship between the taxa, which is why we compare them in this paper for the first time. Clearly, further studies are needed to verify the evolutionary relationships of these two genera. At the present time, we accept the cladogram as is, and both are in the Palaeotriginae.

### Ecological relationships

The most recent phylogenetic analysis of Giraffidae places *Schansitherium* close to *Samotherium* [17]. Our study demonstrates a few similarities between the two taxa, both in cranial and post-cranial material. The main difference between these taxa is in the ossicone structure, however we also observe differences in the premaxillary shape and diet [19]. This pattern is similar to two extant pairs of ruminant genera. *Gazella granti* and *Aepyceros melampus*, and *Damaliscus korrigum* and *Alcelaphus buselaphus*, are two pairs of species from Africa where the main difference is the size and position of the horns. In both of these pairs, the skeletal features are notably similar with only slight variations, and the biggest inter-specific difference arises in the horns [30]. This pattern resembles that of *Schansitherium tafeli* and *Samotherium boissieri*, where there are shared similarities in the skull but the major difference is in ossicone shape and structure. We propose that the difference in ossicone morphology may be linked to difference in ecological function

Interpreting the neck length and ossicone shapes in terms of combat is difficult in extinct animals; however, we offer our hypotheses. The differences in the shape and position of the ossicones of *Schansitherium* and *Samotherium* may suggest varying modes of fighting, however may also be related to varying mating displays or other behavioral differences. For example, the four ossicones of *Schansitherium* may allow the individuals to lock and rotate their heads when fighting, as the modern deer do. This may indicate a difference in the fighting pattern from *Giraffa camelopardalis*, where the long neck is beneficial for a specialized mode of fighting termed “necking.” We hypothesize that the shorter neck of *Schansitherium* may be better suited for fighting involving neck rotation. The ossicones of *Samotherium* resemble the horns of many modern bovids such as those of the gazelles and oryx, which suggests fighting with less rotation and more head-on bashing of the flattened frontal bones [32–33].

Although the sample size of *Schansitherium tafeli* is not large, it is worth noting that all of the ossicones are of approximately the same size. This may indicate that all skulls recovered are male, or more likely, that there is no obvious sexual dimorphism in the ossicones. The lack of gender variation in the cranial appendages is an unusual condition in ruminants. This pattern is seen in the bovid *Pachytagus laticeps* versus *Pachytragus crassicornis* from Samos [34].
